# Left ventricular assist device (LVAD) chronic inflow suck-down: a case report demonstrating the need for retrospective cardiac computed tomography angiography for LVAD assessment

**DOI:** 10.1186/s44348-026-00070-z

**Published:** 2026-03-14

**Authors:** Muhammad Umair, Amy Avakian

**Affiliations:** 1 Department of Radiology, Columbia University, New York, NY USA; 2 Russell H. Morgan Department of Radiology and Radiological Sciences, Johns Hopkins University, Baltimore, MD USA; 3 Southern Hills Hospital and Medical Center, Las Vegas, NV USA

**Keywords:** Left Ventricular Assist Devices, Tomography, X-Ray Computed, Angiography, Electrocardiography-Gated Imaging, Device-Related Complications, Heart Failure

## Background

Left ventricular assist device (LVAD) suction events occur when the inflow cannula contacts the LV endocardium, resulting in reduced inflow cannula flow and potential clinical deterioration. This phenomenon, commonly referred to as “suck-down,” represents dynamic inflow cannula obstruction caused by cyclical myocardial apposition to the cannula orifice.

In this case, a 54-year-old male patient with end-stage heart failure due to ischemic cardiomyopathy, status post implantation of a continuous-flow LVAD (HeartMate 3, Abbott) 4 years prior, presented with recurrent low-flow alarms and intermittent symptoms concerning for mechanical LVAD dysfunction. Given inconclusive echocardiographic evaluation, contrast-enhanced retrospective electrocardiogram (ECG)-gated cardiac computed tomography angiography (CTA) was performed to assess inflow cannula positioning and dynamic obstruction.

Inflow obstruction by LV myocardium can develop from substantial changes in LV size and shape as the ventricle is unloaded by the LVAD; because the LVAD cannula orifice is not surgically anchored, its spatial relationship with LV walls changes over time and throughout the cardiac cycle [1]. Patients with malpositioned inflow cannulas may be predisposed to intermittent inflow cannula obstruction and ventricular arrhythmias from mechanical contact with adjacent endocardium, most commonly the septum [1]. While echocardiography remains the first-line imaging modality for LVAD evaluation, it has inherent limitations due to acoustic shadowing from device components.

**Fig. 1:**
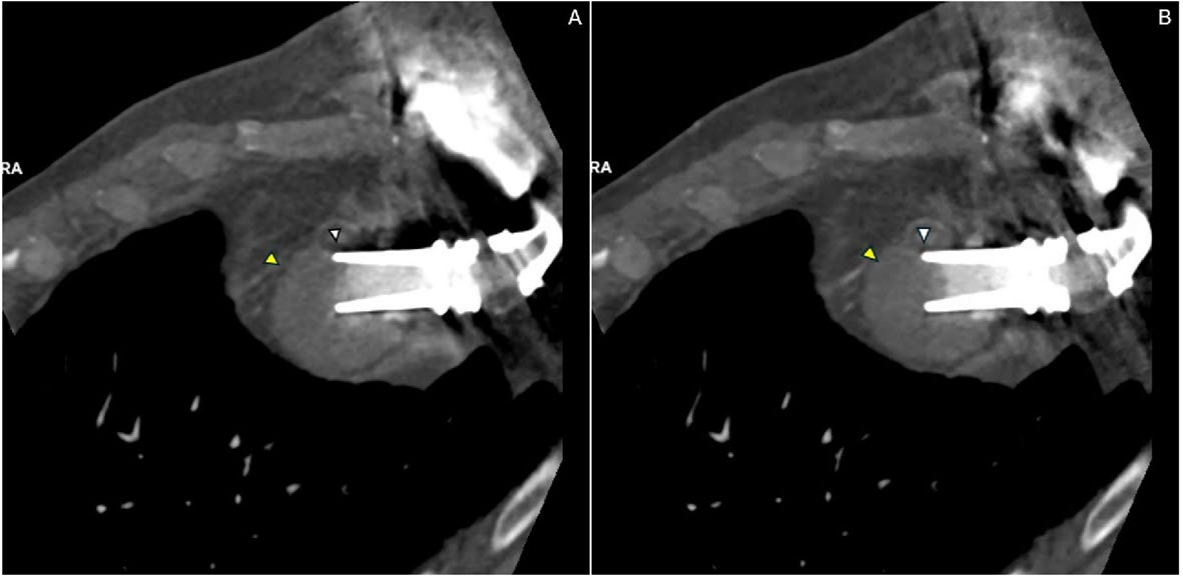
Long-axis multiphasic cardiac computed tomography angiography (CTA) demonstrating dynamic left ventricular assist device (LVAD) inflow cannula obstruction. **A** Diastolic phase multiplanar reformatted cardiac CTA image reconstructed along the long axis of the LVAD inflow cannula demonstrates partial inflow obstruction as the apical interventricular septum abuts the cannula orifice (arrowhead). The inflow cannula orifice is identified by the arrow. This orientation, with the cannula directed toward the interventricular septum rather than parallel to the mitral valve annulus, predisposes to dynamic inflow obstruction. **B** Systolic phase image reconstructed at the same slice level demonstrates progression to complete inflow obstruction as septal myocardium is drawn further into the cannula orifice (arrowhead). The cannula orifice is again identified by the arrow, demonstrating phase-dependent dynamic inflow cannula obstruction (“suck-down”). Images represent matched reconstructions from the same cardiac cycle and slice position to allow direct phase comparison

**Fig. 2:**
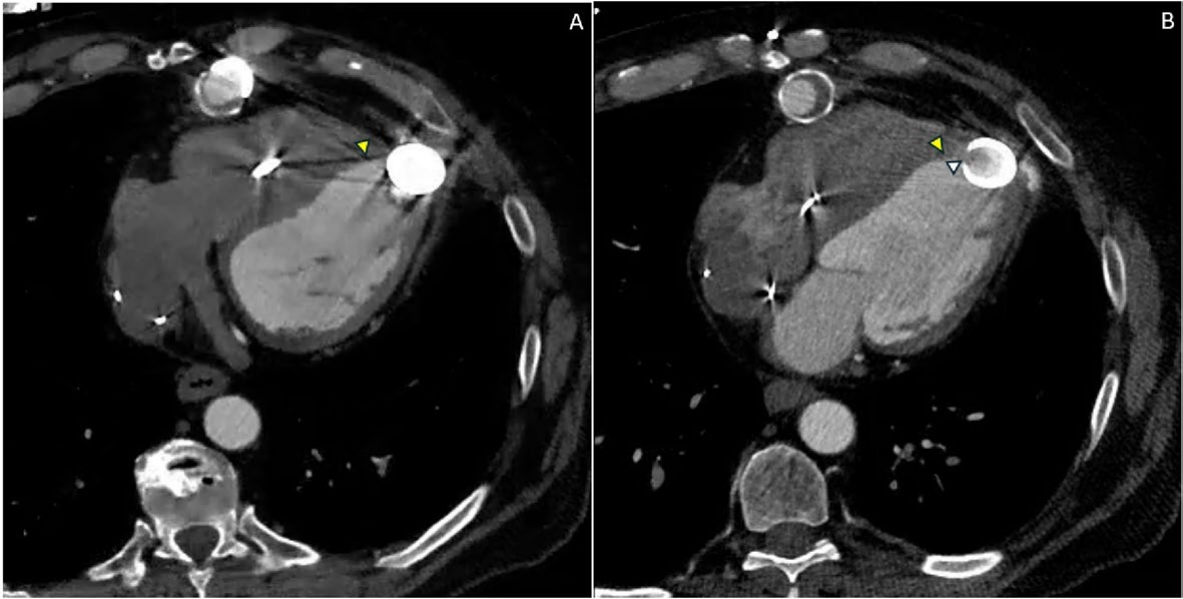
Axial multiphasic cardiac computed tomography angiography (CTA) demonstrating spatial orientation of the left ventricular assist device (LVAD) inflow cannula and phase-dependent septal apposition. **A** Axial diastolic phase cardiac CTA image demonstrates the LVAD inflow cannula (above the level of cannula orifice) directed toward the interventricular septum, with preserved but narrowed inflow channel as septal myocardium contacts the cannula orifice (arrowhead). **B** Axial diastolic phase at a more inferior slice level at the orifice of LVAD inflow cannula demonstrates progression to near-complete inflow obstruction as septal myocardium is drawn deeper into the cannula orifice (arrowhead). The inflow cannula orifice is indicated by the arrow, highlighting the dynamic and cyclical nature of the obstruction

Two-dimensional multiplanar gated cardiac CTA imaging with contrast, with or without 3D reconstruction, is currently the mainstay for evaluation of suspected mechanical LVAD complications [1]. CT can be used to evaluate inflow cannula positioning and for the presence of outflow graft kinking in all contemporary LVADs [2]. ECG gating is essential to avoid motion artifacts, and when information is required to examine dynamic cannula suction, retrospective gating with or without dose modulation is typically used [3]. Cine reconstructions corresponding to the still images are presented in Figs. 1 and 2.

## Discussion

This case demonstrates chronic inflow cannula "suck-down" with dynamic obstruction of the apical interventricular wall. The inflow cannula, which should ideally be directed toward the mitral valve annulus and parallel to the LV outflow tract, was instead oriented toward the interventricular septum [1]. During systole, the septal wall was drawn into the inflow cannula orifice, creating intermittent obstruction that varied throughout the cardiac cycle as shown in Videos 1 and 2. Malpositioning or postoperative displacement of the inflow cannula involving contact with the interventricular septum or LV free wall can lead to suction events, arrhythmias, thrombosis, and hemodynamic compromise [2]. CT-based studies have demonstrated that inflow cannula deviation toward the septum is associated with higher thrombosis risk, with an odds ratio of 1.35 per 10° increase in septal angulation; malposition toward the ventricular septum may contribute to pump thrombosis through a vicious cycle of suction events, low flow, and speed reduction [3].

The dynamic nature of this obstruction underscores the importance of retrospective ECG-gated cardiac CTA rather than prospectively gated or non-gated imaging. Retrospective gating acquires data throughout the entire cardiac cycle, allowing reconstruction at multiple phases to visualize the intermittent nature of septal contact with the inflow cannula [4]. This is particularly critical when dynamic cannula suction is suspected, as static imaging may fail to capture the dynamic obstruction if acquired during a phase when the septum is not in contact with the cannula [4]. Long-axis reconstructions allow direct visualization of phase-dependent septal intrusion into the cannula orifice (Fig. 1), while axial reconstructions provide complementary demonstration of cannula orientation relative to the interventricular septum and confirm the spatial relationship contributing to obstruction (Fig. 2).

Cardiac CT has been shown to improve identification of cardiomechanical LVAD complications when combined with echocardiography, with dynamic obstruction representing the second most common complication identified (26.5% of cases) [5]. The complementary use of both modalities yields superior diagnostic performance compared to either alone, with a combined sensitivity of 67%, specificity of 93%, and diagnostic accuracy of 73% [5]. However, utilizing fully retrospective ECG-gated cardiac CTA, both anatomical and dynamic assessments can be performed in a single examination. This imaging approach may influence LVAD speed optimization, anticoagulation strategies, and consideration of surgical revision.

Compared with prior reports describing acute suction events or static cannula malposition, this case highlights the chronic and cyclical nature of dynamic inflow obstruction and demonstrates the incremental diagnostic value of multiphasic retrospective CTA cine evaluation. The ability to visualize phase-dependent septal apposition to the cannula orifice provides functional insight into obstruction severity and chronicity that cannot be reliably assessed with single cardiac cycle phase imaging or limited-phase acquisition.

## Conclusions

This case demonstrates the need for fully retrospective ECG-gated cardiac CTA for comprehensive assessment of LVAD inflow cannula positioning and dynamic obstruction. Multiplanar and multiphasic cardiac CTA reconstructions provide comprehensive evaluation of LVAD inflow cannula position and dynamic obstruction. Static or prospectively gated imaging may fail to capture intermittent suction events that occur during specific phases of the cardiac cycle. Clinicians should maintain a high index of suspicion for dynamic inflow obstruction in patients with recurrent low-flow alarms or unexplained symptoms and should consider retrospective ECG-gated cardiac CTA as part of the diagnostic evaluation.

## Supplementary Information

Additional file 1: Video 1. Retrospective electrocardiogram (ECG)-gated cardiac computed tomography angiography (CTA) cine loop demonstrating dynamic left ventricular assist device (LVAD) inflow cannula obstruction throughout the cardiac cycle. During diastole, partial obstruction of the inflow cannula is seen as the apical interventricular septum abuts the cannula orifice. During systole, complete obstruction occurs as the septal wall is drawn into the cannula orifice, representing dynamic inflow "suck-down." This cine reconstruction, only possible with retrospective ECG-gated cardiac CTA, demonstrates the cyclical nature of the obstruction that would not be appreciated on static or prospectively gated imaging.

Additional file 2: Video S1. Sequential axial cardiac computed tomography angiography (CTA) images scrolling through the thorax from superior to inferior, demonstrating the anatomic relationship of the left ventricular assist device (LVAD) system to surrounding thoracic structures. The stack demonstrates the outflow graft course, pump housing, and inflow cannula position at the LV apex with orientation toward the interventricular septum.

## Data Availability

No datasets were generated or analysed during the current study.

## Abbreviations

CT: Computed tomography
CTA: Computed tomography angiography
ECG: Electrocardiogram
LV: Left ventricular
LVAD: Left ventricular assist device
